# Online Intelligent Course Education Based on Grid Model Simplification

**DOI:** 10.1155/2022/6009917

**Published:** 2022-07-15

**Authors:** Ning Jin, Zhengkun Yan

**Affiliations:** ^1^Anhui Jianzhu University, Hefei, Anhui, China; ^2^Anhui University of Finance and Economics, Bengbu, Anhui, China

## Abstract

In order to explore the teaching efficiency of online intelligent courses and improve the quality of online teaching, this paper builds a classroom intelligent auxiliary management model based on the grid simplification method. Moreover, this paper formulates corresponding teaching strategies through the recognition of student state features, uses a target detector to detect all detection targets from the scene, and then counts the number of detection targets, identifies specific individuals, and judges the individual state. Simultaneously, this paper intercepts candidate subregions from the scene image and then inputs the subregion image to the detector to determine whether the candidate region image is a detection target and formulate corresponding countermeasures. In addition, on the basis of the existing 3D mesh model stitching and editing method, this paper proposes a grid splicing and fusion method based on the idea of partial reuse of the model to calculate the result of the target. Finally, this paper designs experiments to verify the performance of the model. The research results show that the model constructed in this paper is effective.

## 1. Introduction

With the passage of time and after several years of hard exploration, pilot schools in our country have initially figured out a set of online school-running models. At the same time, a number of online courses and teaching resources have been developed, and an open school pattern has been formed in which multimedia teaching based on the campus network and distance teaching outside the school are carried out simultaneously and are mutually integrated [1]. Therefore, network-based remote multimedia teaching has become a new direction of education reform and development in my country, and it will gradually become the main form of modern remote teaching in China [2]. However, we also clearly see that the current online teaching in China is still in its infancy, and the commonly used teaching method is to set up teaching stations in concentrated areas of students. For example, some prestigious schools set up teaching sites across the country. The non-real-time network courseware teaching and the scattered and isolated remote sites of universities hinder the large-scale sharing and exchange of online educational resources [3]. Online distance education means, with the guidance of modern education thought and learning theory, to provide educators and learners with an online teaching and learning environment to deliver digital content and to develop learner-centered non-face-to-face education activities under the network environment, which gives full play to the various educational functions of computer networks and the advantages of rich network education resources [4]. With the popularization and development of computer networks, computer network distance education is playing an increasingly important role [5].

As a new teaching method, online teaching makes full use of the latest computer technology, network technology, and multimedia technology so that the teaching process can cross the limitations of time and space, with great flexibility and interactivity [6]. Moreover, online teaching makes the display of teaching materials more and more flexible and vivid. In addition to the ordinary functions of traditional teaching, online teaching also has functions that cannot be achieved by traditional face-to-face teaching in some aspects, such as answering questions and discussing [7]. In traditional education, due to psychological effects and time and space constraints, there are limitations in the answering and communication between teachers and students. However, online teaching can easily overcome psychological barriers, overcome time and space constraints, and teachers and students can communicate in various ways. A large number of exchanges between teachers and students have a subtle effect on the implementation of research teaching and the cultivation of creative talents. Based on these data, an objective evaluation of the teaching effect can be made [8]. Through the preservation, statistical analysis, and data mining of these data, useful information can be provided for teaching activities and teaching decisions [9].

## 2. Related Work

The literature proposed a user-assisted mesh simplification algorithm based on vertex merging, which provides the possibility to independently simplify each subobject at different levels of detail [10]. It is worth pointing out that this method can complete the simplification and refinement of the required subobjects and can be executed while maintaining the total number of triangles in the simplified model. The literature proposed a simplified multilateral extraction algorithm aimed at improving the transmission efficiency of three-dimensional objects, which is achieved by constructing a simple triangle containing the information of the internal segmentation vertices [11]. The literature also proposed a new mesh simplification algorithm based on edge simplification, which decouples the simplification process into two stages: shape analysis and edge shrinking [12]. The simplified algorithm proposed in the literature is implemented by triangle extraction [13]. This literature took human vision into consideration, introduced a metric based on perception, and proposed an efficient simplified algorithm based on the human visual system. The literature proposed a tetrahedral simplification method based on triangle extraction. There are also many ways to simplify the process measurement method [14]. The literature proposed a feature-preserving mesh simplification algorithm based on volume measurement, which combines the square volume error of the triangle, the shape factor, and the normal constraint factor to define the simplification error and minimize the error objective function [15]. The literature proposed a method of discrete curvature error measurement to generate level of detail (LOD). The edge folding algorithm is the most widely used 3D mesh simplification algorithm [16]. This algorithm is also the basic algorithm for the follow-up research of the article, and most of the grid simplification algorithms are to simplify the grid through edge transformation.

## 3. Algorithm and Model Design

### 3.1. A Generation Algorithm Based on Machine Learning Candidate Regions

In RCNN, the selective search algorithm is used to generate candidate images. This algorithm first generates a number of oversegmented small regions and then merges the small regions according to the similarity, so the computational complexity is relatively large. The biggest problem with the selective search algorithm's slow calculation speed is the image oversegmentation and merging process. Oversegmentation is a process of finding a certain similar area based on a random location pixel as the center, which divides the image into small areas according to the pixel gray value. The application scenarios of crowd statistics algorithms are often complicated and changeable due to interference, such as illumination, diversity of human clothing, and occlusion. Moreover, the candidate regions generated by the selective search algorithm are often irregular and fragmented either a candidate region is too small to contain a complete human body or a candidate region is too large to contain multiple human bodies.

In the improved candidate region generation algorithm, the gray world algorithm is first used to perform white balance preprocessing on the preprocessed image to reduce the impact of illumination on the scene image. Furthermore, the white balance preprocessing method of the gray world algorithm first averages the three channels of the preprocessed image, then calculates the gain of each channel and superimposes the gain value on the original image, and finally performs planning processing on the result. The white balance processed image will automatically equalize the gray value of the pixels to prevent the overall image from being bright or dark and to remove the interference of light to a certain extent. The formula is as follows:

(1) $$\begin{array}{c} \alpha =\frac{\left(M_{R}+M_{G}+M_{B}\right)}{3}, \end{array}$$

(2) $$\begin{array}{c} K=\left(\frac{\alpha }{M_{R}},\frac{\alpha }{M_{G}},\frac{\alpha }{M_{B}}\right), \end{array}$$

(3) $$\begin{array}{c} R_{\text{new}}=R\cdot \frac{\alpha }{M_{R}}, \end{array}$$

(4) $$\begin{array}{c} B_{\text{new}}=B\cdot \frac{\alpha }{M_{B}}, \end{array}$$

(5) $$\begin{array}{c} I_{\text{out}}=\left(R_{\text{new}},G_{\text{new}},B_{\text{new}}\right). \end{array}$$

Among them, *M*_*R*_, *M*_*G*_,  and *M*_*B*_ represent the average value of the three channels of the input image *R*, *G*, and B, *α* represents the global average value of the three channels, K represents the gain value of each channel, *R*_new_, *G*_new_,  and *B*_new_ represent the three channels after adding the gain, and *I*_out_ represents the image after gain superposition. Regarding the possible overflow phenomenon in the abovementioned processing, after many experiments, the results show that if the calculated value of pixels greater than 255 is directly set to 255, it is easy to cause the image to be globally whitened. However, the method of using the maximum value of each channel of *R*_new_, *G*_new_,  and *B*_new_ as the upper limit to linearly compress all values into [0,255] can achieve the effect of equalizing grayscale.

Then, the background segmentation method of the K nearest neighbor algorithm (KNN) is used to extract the preprocessed image. The method is to traverse the input image, find the K pixels closest to each pixel in a certain neighborhood, vote on the category of these points, determine whether the pixel is background or foreground, and update the background. Each frame of the video is divided into background and foreground. The classification decision rules are as follows:

(6) $$\begin{array}{c} y=\arg\underset{c_{j}}{\max}\sum_{x_{i}\in N_{k}\left(x\right)}I\left(y_{i}=c_{j}\right),\\ i=1,2,\text{…},N;j=1,2,\text{…},K. \end{array}$$

Among them, *I*(·) is the indicator function, that is, the function is 1 when *y*_*i*_=*c*_*j*_; otherwise, the function is 0.

Then, the dilation and erosion operation is performed on the extracted moving foreground to eliminate noise and obtain the final foreground area.

Finally, the extracted image is detected by a method that guarantees the invariance of perspective, and the detected image pixel coordinates (*x*, *y*) are input into the trained linear model to obtain the size of the human body area.

Furthermore, we use the sliding window method to traverse each pixel in the foreground area and then input the coordinate (*x*, *y*) of each pixel point traversed into the human body region model to obtain the size of the human body region. Moreover, we adopt the perspective and perspective invariance and use the linear regression model to establish the relationship between the spatial coordinates of the fixed scene image pixel and the size of the human body area. Before training, we manually intercept the human body area at each position from the scene to cover all the coordinates from far and near as much as possible. Then, we use linear regression to train the model to get the human body region model. The formula is as follows:

(7) $$\begin{array}{c} J\left(\theta \right)=\frac{1}{2}\sum_{l=1}^{n}{\left(h_{\theta }\left(x^{i}\right)-y^{i}\right)}^{2},\\ \theta_{i}=\theta_{i}-\alpha \frac{\partial }{\partial \theta }J\left(\theta \right)=\theta_{i}-\alpha \left(h_{\theta }\left(x\right)-y\right)x^{i}. \end{array}$$

Among them, formula (1) is the objective function, *h*_*θ*_(*x*) represents the linear estimation function of the target problem, *y* represents the true value of the target problem, formula (2) is the weight update function, *θ* represents the weight of the linear model, and *α* represents the learning rate.

Specifically, each traversed pixel coordinate (*x*, *y*) is taken as the center of the subregion, and the subimage is taken from *I*_out_ as the image to be detected. The calculation formula for the size of the human body area is as follows:

(8) $$\begin{array}{c} w=\theta_{0}+\theta_{1}\cdot x+\theta_{2}\cdot y,\\ h=\omega_{0}+\omega_{1}\cdot x+\omega_{2}\cdot y,\\ \theta =\left[\begin{array}{c} \theta_{0}\\ \theta_{1}\\ \theta_{2} \end{array}\right],\\ \omega =\left[\begin{array}{c} \omega_{0}\\ \omega_{1}\\ \omega_{2} \end{array}\right]. \end{array}$$

**Figure 1:**
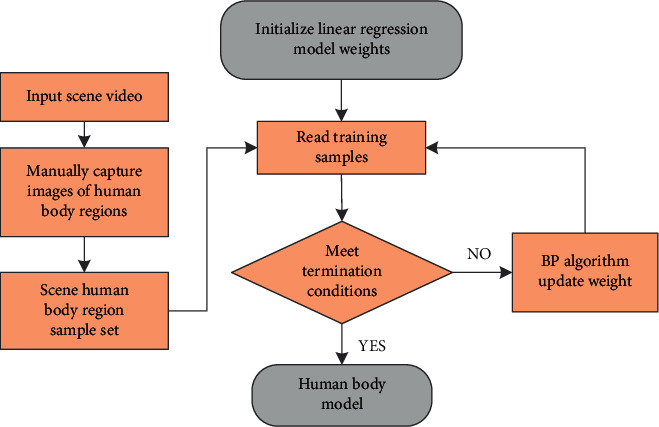
Model training process.

Among them, *w* and *h*, respectively, represent the width and height of the human body area at the coordinates (*x*, *y*). *θ* and *ω*, respectively, represent the weights of the linear model for finding the width and height of the human body region, and *θ*_*i*_ and *ω*_*i*_ are the learnable weights, which are obtained by manually intercepting the human body region from the detection scene and training using a linear regression algorithm. The training process of the entire candidate area generation algorithm is shown in Figure 1.

Since the perspective relationship between the distance and size of the object is a linear relationship, the calculation of the human body size is based on the human body region model of linear regression. Using machine learning methods to ensure the invariance of perspective and perspective makes it easier to recalibrate the system after installing and deploying the system or changing the camera's shooting angle and shooting distance.

### 3.2. Algorithm Design and Implementation

This section introduces the crowd statistics algorithm based on deep learning. This method proposes an algorithm that uses a sliding window to intercept candidate regions, then extracts the integration channel features, and uses a cascade classifier to determine the heads to count the number of people. This method of counting the population depends very much on the quality of the detection. The detector detects all the heads in the image and then counts the number of people in the scene.

The technical route of the crowd statistics algorithm based on deep learning is to use the gray world algorithm for image white balance preprocessing and then use the background segmentation method of the K nearest neighbor algorithm to extract the preprocessed image. Next, it uses a method to ensure the invariance of viewing angle and perspective to detect the extracted image and input the detected image pixel coordinates (*x*, *y*) into the trained linear model to obtain the size of the human body area. Finally, it uses a convolutional neural network as a human body monitoring model to detect the human body in the candidate area and finally counts the number of regions judged to be a human body as the statistical result.

**Figure 2:**
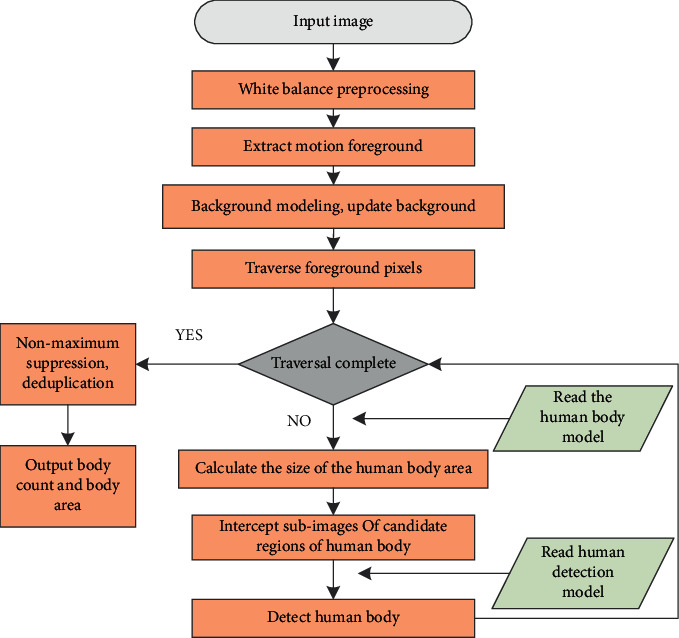
The process of crowd statistics algorithm based on deep learning.

The overall process of the statistical algorithm can be divided into three stages: candidate area generation, target detection stage, and people counting stage. The improved algorithm introduced in this chapter is named “crowd statistics algorithm based on deep learning, ” and the overall flow chart is shown in Figure 2.

The specific implementation process is as follows:

(1) The stage of candidate region generation
  Input: training image data sequence {*I*_1_, *I*_2_, ⋯, *I*_*t*_, ⋯} and candidate region model [*θ*, *ω*].
  Output: subimage sequence {*S*_1_, *S*_2_, ⋯, *S*_*t*_, ⋯} of the candidate area.
  (1) The gray world algorithm is used to preprocess the white balance of *I*_*t*_ to obtain the image *S*_*t*_.
  (2) The background segmentation method of the K nearest neighbor algorithm is used to extract the foreground mark *F*_*t*_ in image *S*_*t*_.
  (3) The method of ensuring the perspective and perspective invariance is used to slide in the image *F*_*t*_, and a certain pixel coordinate (*x*, *y*) marked as a foreground pixel is input into the trained candidate area model [*θ*, *ω*] to obtain the size *Size*_*t*,*k*_ of the candidate area. The calculation formula is as follows:
    (9) $$\begin{array}{c} w=\theta_{0}+\theta_{1}\cdot x+\theta_{2}\cdot y,\\ h=\omega_{0}+\omega_{1}\cdot x+\omega_{2}\cdot y. \end{array}$$
  (4) The algorithm continues to perform step 3 until all the foreground pixels are traversed to obtain the coordinates and size {*R*_1_, *R*_2_, ⋯, *R*_*n*_, ⋯} of all candidate regions, and all candidate region image sequences {*S*_1_, *S*_2_, ⋯, *S*_*n*_, ⋯} are cut out from the scene image *I*_*t*_.
(2) Object detection stage
  Input: image sequence {*S*_1_, *S*_2_, ⋯, *S*_*n*_, ⋯} of the candidate area and human body monitoring model *F*(·)
  Output: human body detection result {*L*_1_, *L*_2_, ⋯, *L*_*n*_, ⋯} in candidate area image.
  The candidate area image sequence is input into the deep network to detect the human body, and the calculation formula is as follows:
    (10) $$\begin{array}{c} F\left(\left(\begin{array}{c} S_{1}\\ \vdots \\ S_{n} \end{array}\right)\right)=\left(\begin{array}{c} L_{1}\\ \vdots \\ L_{n} \end{array}\right). \end{array}$$
(3) People counting stage
  Input: the coordinates and size {*R*_1_, *R*_2_, ⋯, *R*_*n*_, ⋯} of the selected area, the human body detection result is {*L*_1_, *L*_2_, ⋯, *L*_*n*_, ⋯}
  Output: the number of person_count

The nonmaximum suppression algorithm is used for all subregions judged to be human in the detection result to remove redundant regions. All regions determined to have a human body are sorted according to the network output value, that is, the confidence level of the human body. Then, the region with the highest confidence is used as the standard, and all regions that exceed a certain set threshold are removed. The formula is as follows:

(11) $$\begin{array}{c} o=\frac{S_{\text{over}}}{S},\\ f\left(o\right)=\left\{\begin{array}{cc} 0, & o>\sigma ,\\ 1, & o\leq \sigma . \end{array}\right. \end{array}$$

Among them, *S*_over_ represents the area of the overlap of the two regions participating in the determination, S represents the total area of the two regions participating in the determination, and *o* represents the proportion of the overlap of the area in the total area. The area where *f*(*o*) is 0 is removed, and the remaining area is the final result. According to experiments, the method achieves the best effect when *σ*=0.2.

### 3.3. Algorithm Complexity Analysis

Based on the crowd statistics algorithm of the human detection network, the KNN motion foreground is extracted first, and the input image is assumed to be *N* × *N*. KNN nearest neighbor background modeling will compare the current frame and the previous K frame image at the same pixel Euclidean distance pixel-by-pixel to determine whether the pixel belongs to the background. Therefore, the computational complexity is

(12) $$\begin{array}{c} O\left(K\cdot N^{2}\right). \end{array}$$

The sliding window algorithm traverses the foreground pixels and intercepts candidate subregion images and inputs them into the human body detection network. If the size of the candidate region of the human body is *n* × *n*, the number of candidate regions captured by the sliding window is

(13) $$\begin{array}{c} \left[\frac{N-n}{\text{Stride}}\right]. \end{array}$$

The candidate images of *n* × *n* are input into the network classification. The human body detection network has two convolutional layers, two pooling layers, two regularization layers, and two fully connected layers.

In general, the input and output feature maps of the 2D convolutional layer have the same length and width. The width of the output feature map is *M* × *M*, the size of the 2D convolution kernel is *K* × *K*, the number of input feature map channels of the network is *C*_in_, and the number of output feature map channels of the network is *C*_out_. After determining the padding and stride hyperparameters, we know that

(14) $$\begin{array}{c} M=\frac{\left(n-K+2\cdot \text{padding}\right)}{\text{stride}+1}. \end{array}$$

Therefore, the computational complexity of the 2D convolutional layer is

(15) $$\begin{array}{c} O\left(M^{2}\cdot K^{2}\cdot C_{\text{in}}\cdot C_{\text{out}}\right). \end{array}$$

The input feature map size of the max pooling layer is *N* × *N*, the number of channels is C, and the size of the convolution kernel is *K* × *K*. The size of the output feature map is

(16) $$\begin{array}{c} \frac{N}{2}. \end{array}$$

Traverse the pixels in the subregion, calculate the maximum value, complexity: *K*^2^.

Then, the computational complexity is

(17) $$\begin{array}{c} C\cdot \frac{N^{2}}{2}\cdot K^{2}. \end{array}$$

Usually, *K*=2, so usually max pooling layer complexity is

(18) $$\begin{array}{c} O\left(C\cdot N^{2}\right). \end{array}$$

The function of the LRN layer is to regularize the pixel-level historical value of the feature map. Because when we want to normalize the output of the LRN layer at time *T* before, we need to accumulate the square of the output value of the LRN layer at time *T* before, and we set the input feature map as *N* × *N* and the number of channels as C. The computational complexity is

(19) $$\begin{array}{c} O\left(C\cdot T\cdot N^{2}\right). \end{array}$$

Finally, in the fully connected layer, the input feature is *S* × *M*_in_*n* and the output feature is *S* × *M*_out_. Then, the dimension of the weight matrix is *M*_in_ × *M*_out_. Therefore, according to the matrix operation rules, the computational complexity of the fully connected layer is

(20) $$\begin{array}{c} O\left(S\cdot M_{\text{in}}\cdot M_{\text{out}}\right). \end{array}$$

## 4. Model Building

**Figure 3:**
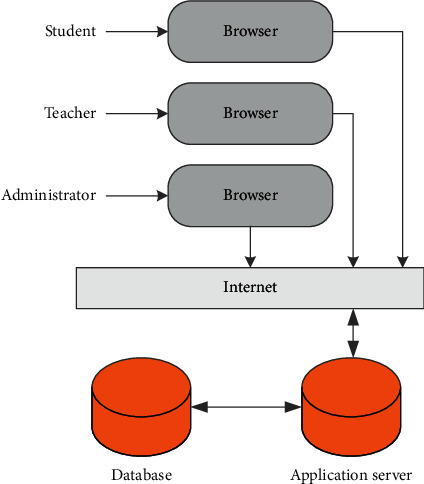
Internet-based remote teaching mode.

This system is based on the Internet. The system is designed for remote teaching. All functions are completed through the interaction between the application server and the user's browser. The data related to the system is organized and maintained by the database server.  Figure 3 is a simple schematic diagram of the system teaching mode. Teachers, students, and administrators connected through the Interact network send requests to the application server from the browser. The application server responds accordingly to the user's request, retrieves data from the database server when necessary, and feeds back the service result to the user through the browser.

**Figure 4:**
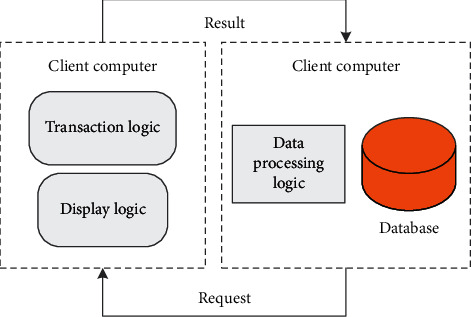
Two-tier CS architecture diagram.

The C/S model is mainly composed of two major components: client and server. Display logic and transaction processing logic are placed on the client, and the client is responsible for completing the foreground functions, such as managing user interfaces, data processing, and report requests. The data processing logic and database are placed on the server, and the server part performs background services and responds to client requests, such as managing shared peripherals, controlling the manipulation, and acceptance of shared databases, as shown in Figure 4.

This traditional two-tier C/S architecture is more suitable for small-scale operation in a local area network environment with fewer users, a single database, and security and speed. Therefore, it has been widely used under the current conditions. However, with the large-scale application system and the continuous improvement of users' requirements for system performance, the two-tier C/S structure is increasingly unable to meet the higher needs of users. This is mainly reflected in the fact that each client must run the same logic, resulting in a serious waste of resources, multiple clients connecting to the database easily cause network congestion, and program maintenance is particularly difficult.

**Figure 5:**
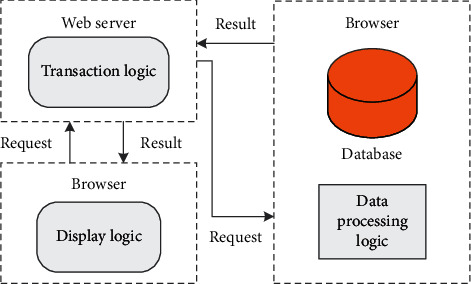
Three-tier B/S architecture diagram.

With the enhancement of browser functions, a new type of architecture B/S emerged and developed rapidly. The B/S structure greatly simplifies the work of the client. The client only needs to install and configure a small amount of client software. The server will take on more work. The access to the database and the execution of the application program are only completed by the server. Its architecture is shown in Figure 5.

The layers of the three-tier B/S architecture are independent of each other, and changes in any one layer do not affect the functions of other layers. It fundamentally changes the defects of the traditional two-tier C/S structure, and it is a profound change in the application system architecture. For distance education systems, the use of C/S architecture is definitely not the best choice. The nature of network computer distance education determines that it is impossible for students to come to school and concentrate on the onsite lecture. In fact, since the students can be said to be distributed all over the world, it is impossible to install and maintain a client program for the students. Therefore, we decided to adopt the form of web service and set up a virtual classroom system with B/S architecture to meet the needs of practical applications.

**Figure 6:**
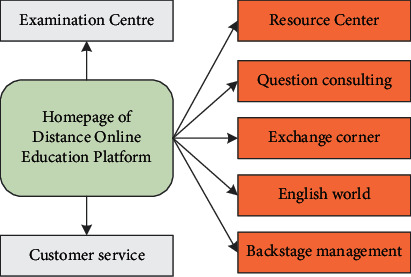
Model structure diagram.

According to actual needs, this paper constructs a platform that can be applied to distance network education, and its structure is shown in Figure 6.

**Figure 7:**
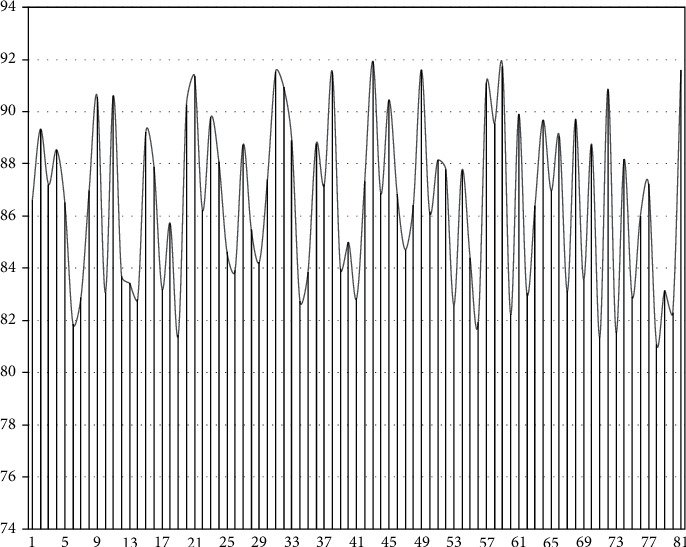
Statistical diagram of the accuracy of student feature recognition.

After constructing the above model, this paper verifies the performance of the model. The model in this paper uses the grid model to identify students online, thereby judging the status of students. Combining actual needs, this paper mainly conducts research through model feature recognition performance verification and curriculum teaching decision-making effect in performance evaluation. First, this paper analyzes the effect of student feature recognition, recognizes the features of 81 students in a certain major, and compresses with the actual situation to obtain the accuracy of feature recognition. The results are shown in Figure 7.

**Figure 8:**
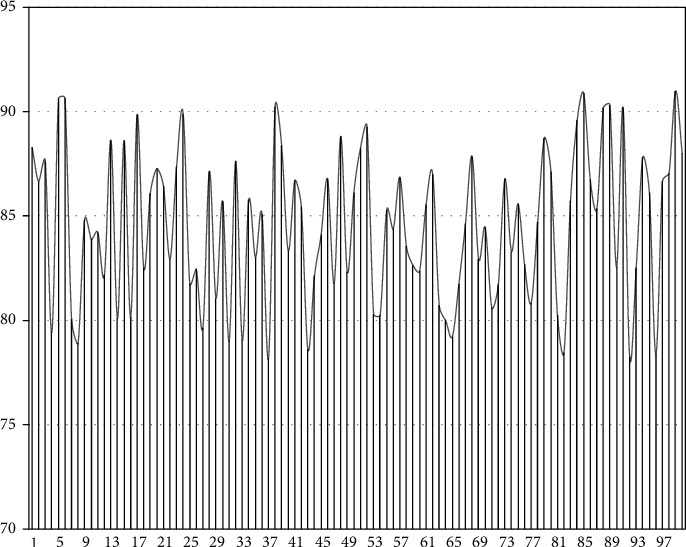
Statistical diagram of auxiliary teaching effect score.

As shown in Figure 7, the model in this paper has a high accuracy rate for student feature recognition. Next, the auxiliary teaching effect after model recognition is scored, and 100 groups are evaluated. The results are shown in Figure 8.

As shown in Figure 8, the auxiliary teaching effect of this paper is relatively good, so it can be seen that the model constructed in this paper meets the expected effect.

## 5. Conclusion

This paper combines the grid model simplification method to construct an online intelligent course teaching model and formulates corresponding teaching strategies through the recognition of student status characteristics. The student statistical algorithm based on deep learning uses the gray world algorithm to do image white balance preprocessing and then uses the background segmentation method of the K nearest neighbor algorithm to extract the preprocessed image. Next, it uses methods to ensure the invariance of perspective and perspective to detect the extracted images and counts the number of regions determined to be human bodies as the statistical result. In addition, on the basis of the existing 3D mesh model splicing editing method, this paper uses the idea of partial reuse of the model to propose a mesh splicing and fusion method using cubic B-spline interpolation. This method can well handle the smoothness of the splicing area when the models are spliced and obtain a good splicing effect. The experimental results also prove that the model has a certain effect.

## Data Availability

The data used to support the findings of this study are available from the corresponding author upon request.
